# Short-term versus long-term mentalization-based therapy for outpatients with subthreshold or diagnosed borderline personality disorder: a protocol for a randomized clinical trial

**DOI:** 10.1186/s13063-019-3306-7

**Published:** 2019-04-05

**Authors:** Sophie Juul, Susanne Lunn, Stig Poulsen, Per Sørensen, Mehrak Salimi, Janus Christian Jakobsen, Anthony Bateman, Sebastian Simonsen

**Affiliations:** 1 Stolpegaard Psychotherapy Centre, Mental Health Services, Gentofte, Capital Region of Denmark Denmark; 2 Department of Psychology, University of Copenhagen, Copenhagen, Denmark; 3 Copenhagen Trial Unit, Center for Clinical Intervention Research, Rigshospitalet, Copenhagen University Hospital, Copenhagen, Denmark; 4 St. Anns Hospital, London, England

**Keywords:** Mentalization-based therapy, Borderline personality disorder, Randomized clinical trial, Treatment intensity

## Abstract

**Background:**

Psychotherapy for borderline personality disorder is often lengthy and resource-intensive. However, the current length of outpatient treatments is arbitrary and based on trials that never tested if the treatment intensity could be reduced. As a result, there is insufficient evidence to inform the decision between short-term and long-term psychotherapy for borderline personality disorder. Mentalization-based therapy is one treatment option for borderline personality disorder and consists traditionally of an 18-month treatment program.

**Methods/design:**

This trial is an investigator-initiated single-center randomized clinical superiority trial of short-term (20 weeks) compared to long-term (14 months) mentalization-based therapy for outpatients with subthreshold or diagnosed borderline personality disorder. Participants will be recruited from the Outpatient Clinic for Personality Disorders at Stolpegaard Psychotherapy Centre, Mental Health Services, Capital Region of Denmark. Participants will be included if they meet a minimum of four DSM-V criteria for borderline personality disorder. Participants will be assessed before randomization, and at 8, 16, and 24 months after randomization. The primary outcome is severity of borderline symptomatology assessed with the Zanarini Rating Scale for borderline personality disorder. Secondary outcomes include self-harm incidents, functional impairment (Work and Social Adjustment Scale, Global Assessment of Functioning) and quality of life (Short-Form Health Survey 36). Severity of psychiatric symptoms (Symptom Checklist 90-R) will be included as an exploratory outcome. Measures of personality functioning, attachment, borderline symptoms, group alliance, and mentalization skills will be included to explore potential predictors and mechanisms of change.

**Discussion:**

This trial will provide evidence of the beneficial and harmful effects of short-term compared to long-term mentalization-based therapy for outpatients with subthreshold or diagnosed borderline personality disorder.

**Trial registration:**

ClinicalTrials.gov, NCT03677037. Registered on September 19, 2018.

**Electronic supplementary material:**

The online version of this article (10.1186/s13063-019-3306-7) contains supplementary material, which is available to authorized users.

## Background

Borderline personality disorder is a psychiatric condition characterized by a pervasive pattern of symptoms such as interpersonal conflicts, identity diffusion, impulsivity, and emotional dysregulation [1]. According to epidemiological studies, 1.6% of the general population suffer from borderline personality disorder [2]. In clinical populations, it is the most common personality disorder [2], with a prevalence of between 9% and 22% of all psychiatric outpatients [3–5]. Borderline personality disorder is associated with high levels of psychiatric comorbidity, particularly depression, anxiety disorders, eating disorders, substance abuse [6–8], and other personality disorders [9]. Together, these findings emphasize the need for the development of efficacious and cost-effective treatments for this severe and highly prevalent disorder.

While pharmacological treatment may reduce some borderline-related symptoms, there is still no convincing evidence that it is suitable for treating all diagnostic criteria [10]. Although further evidence is still warranted, psychotherapy continues to be the primary treatment of choice for borderline personality disorder [11]. During the last 10–15 years, studies have established the efficacy of different forms of intensive, specialized long-term psychotherapy modalities. These have recently been evaluated in a systematic review and meta-analysis exploring the efficacy of psychotherapies for borderline personality disorder, in which it was concluded that dialectical behavior therapy and psychodynamic therapies (transference focused therapy and mentalization-based therapy) significantly improved borderline-relevant outcomes [12]. However, no single treatment modality has been established as the primary treatment of choice.

Mentalization-based therapy (MBT) is a psychodynamic therapy rooted in attachment and cognitive theory [13], which was developed specifically for treating borderline personality disorder [14]. Mentalization refers to the capacity to understand one’s own and others’ mental states. The theoretical assumption is that patients with borderline personality disorder are more vulnerable to lose this capacity when experiencing emotional distress. The MBT manual offers therapeutic techniques to identify these shifts and to bring the patient back into a mentalizing mode [14, 15]. The therapy program consists of four basic components: (1) psycho-education, (2) case formulation, (3) group therapy, and (4) individual therapy. All of these aim to enhance the patient’s capacity to mentalize. Increasing mentalization skills is assumed to minimize borderline-related symptoms such as emotional dysregulation, impulsivity, and suicidal ideation. However, information about the mechanisms that produce a change in MBT, or in psychotherapy in general, is still limited [16, 17].

MBT for adult borderline personality disorder has been tested in cohort studies [18, 19] and one randomized but uncontrolled trial [20]. Two forms of MBT have been tested in randomized controlled trials: day hospital MBT [21, 22] and intensive outpatient MBT [13], each lasting a maximum of 18 months. For a systematic review of the current evidence base of MBT for borderline personality disorder, see Vogt and Norman [16].

Bateman and Fonagy [13] assessed the effects of intensive outpatient MBT in a randomized clinical trial, in which 134 participants with a confirmed borderline personality disorder diagnosis were randomized either to 18 months of outpatient MBT, combining weekly group and individual sessions with different therapists, or to structured clinical management. In this trial, MBT was superior to structured clinical management in terms of its effects on suicide attempts, severe incidents of self-harm, and on self-reported measures. Treatment effects were sustained at the 5-year follow-up [23]. Nevertheless, only 134 participants were randomized, which questions whether the trial was powered to assess the chosen outcomes, and only 41 were assessed after 5 years. Further, the trial investigators were also the developers of MBT. Thus, the small sample size and the substantial problems with incomplete outcome data, especially at the long-term follow-up, are threats to the validity of the study.

However, while intensive outpatient MBT currently has empirical support as an 18-month program for borderline personality disorder, evidence that this is the optimal length of the intervention is not available. Consequently, MBT is now offered for different lengths of time (both shorter and longer) in outpatient settings around the world [14]. Various other short-term psychotherapies for borderline personality disorder have already been developed and tested in randomized clinical trials, e.g., emotion regulation group therapy [24], systems training for emotional predictability and problem-solving [25, 26], and brief dialectical behavior therapy skills training [27]. However, all the trials have either compared a short-term experimental group to a short-term control group or tested the short-term treatment as an adjunctive to treatment as usual. Thus, these trials do not provide guidance on evidence-based decisions regarding the optimal length of treatment for borderline patients. In addition, no empirical evidence is available to identify which subtypes of patients would benefit from short-term treatment and which would require more intensive treatment [28].

We performed a preliminary literature search (PubMed and Cochrane Library) for trials comparing different lengths of psychotherapy for borderline personality disorder. No such trials were found. When we expanded our search terms to all types of psychiatric disorders, only few trials were identified [29, 30]. We are currently working on a protocol for a more comprehensive systematic review, including a full assessment of risk of bias and a trial sequential analysis of short-term compared with long-term psychotherapy for all psychiatric disorders. The systematic review will be submitted for publication before data collection is completed in this trial.

## Methods/design

### Objective

The primary objective of this trial will be to evaluate the beneficial and harmful effects of short-term (20 weeks) MBT compared with long-term (14 months) MBT for adult outpatients with subthreshold or diagnosed borderline personality disorder. We will evaluate the treatments on the primary outcome (borderline symptomatology), secondary outcomes (self-harm incidents, quality of life, and functional impairment, and exploratory outcomes (psychiatric symptoms). Measures of personality functioning, attachment, borderline psychopathology, group cohesion, and mentalization skills will be included as predictor and mediator variables.

### Design

**Fig. 1:**
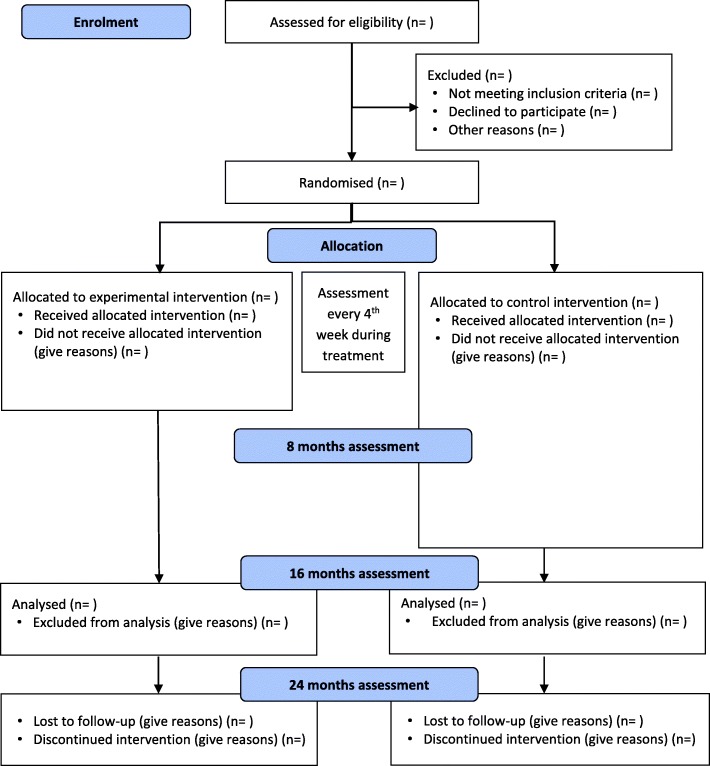
Consolidated Standards of Reporting Trials (CONSORT) flow chart

**Fig. 2:**
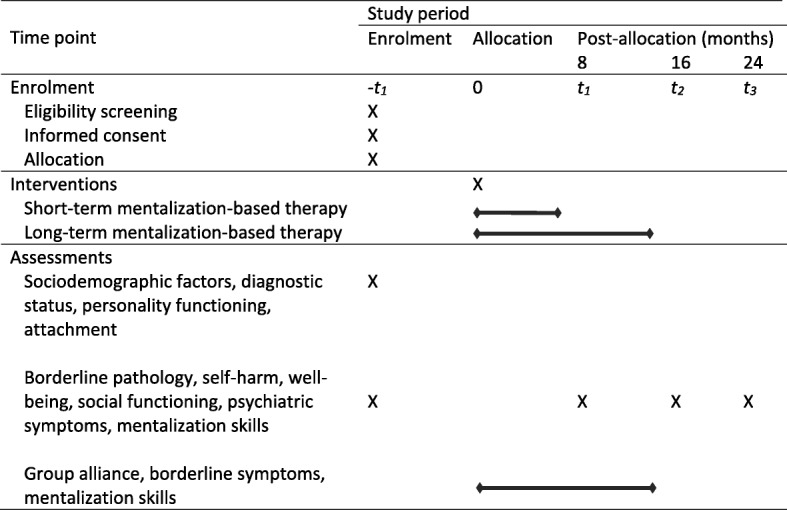
Participant timeline for the Standard Protocol Items: Recommendations for Interventional Trials (SPIRIT)

We have designed an investigator-initiated parallel-group single-centre randomized clinical superiority trial of short-term versus long-term MBT for outpatients with subthreshold or diagnosed borderline personality disorder. The Consolidated Standards of Reporting Trials (CONSORT) flow chart for the trial is shown in Fig. 1. [31, 32]. The Standard Protocol Items: Recommendations for Interventional Trials (SPIRIT) participant timeline is given in Fig. 2, and the SPIRIT checklist is given in Additional file 1 [33].

We will consider for participation all patients referred to the trial site. Patients will be included in the trial, if they comply with the eligibility criteria listed in Table 1. There are inclusion and exclusion criteria as part of the procedure for clinical intake at the trial site, and criteria specific to this trial. For a detailed overview of typical patient characteristics at the trial site, see Simonsen et al. [34].

**Table 1:** Eligibility criteria

	Criteria exclusive to the outpatient clinic	Criteria exclusive to the trial
Inclusion criteria	Aged 18–60 Personality disorder(s) considered to be the primary diagnosis/diagnoses	A minimum of four confirmed DSM-V diagnostic criteria for borderline personality disorder Written informed consent
Exclusion criteria	Possibility of a learning disability (IQ < 75) A full diagnosis of schizotypal personality disorder or antisocial personality disorder Presence of a comorbid psychiatric disorder that requires specialist treatment Current (past 2 months) substance dependence including alcohol Concurrent psychotherapeutic treatment outside the clinic	Unable to understand Danish Lack of informed consent

*DSM-V* Diagnostic and Statistical Manual of Mental Disorders, 5th edition

We will include participants with at least subthreshold borderline personality disorder. According to the Diagnostic and Statistical Manual of Mental Disorders, 5th edition (DSM-V) [1], the threshold for a full diagnosis is five out of nine diagnostic criteria. However, there is increasing evidence that even four confirmed diagnostic criteria are not qualitatively different from diagnosed borderline personality disorder in terms of impairment, and that the diagnostic threshold should be more inclusive than established by the DSM system to reflect the dimensionality of the construct [35, 36]. For this reason, previous trials have included participants with at least a subthreshold diagnosis [24, 37], and we will do the same in this trial.

### Trial site and personnel

The trial site is the Outpatient Clinic for Personality Disorders at Stolpegaard Psychotherapy Centre, Mental Health Services, Capital Region of Denmark (from now on referred to as “the clinic”). The clinic specializes in MBT for borderline personality disorder. Patients are referred from the Capital Region of Denmark via a central visitation unit, where they are initially screened for eligibility before referral to the clinic. Once referred to the clinic, psychiatrists and attending physicians will perform the initial selection and screening of a participant to the trial and collect informed consent. The principal investigator, sponsor-investigator, or a trained research assistant will then conduct the baseline assessments of the participant. All post-baseline assessments will be carried out by trial investigators who are blind to treatment allocation.

Trial therapists provide therapy to both the short-term and long-term treatment groups. Before commencing the trial, all trial therapists at the clinic will have received training in the short-term MBT program by trial investigators and national and international MBT specialists. The training covers relevant topics like case formulations, termination of psychotherapy, and case-specific supervision. The training will continue throughout the trial period. Therapist treatment fidelity will be rated by an independent certified rater. This will allow us to investigate whether the delivered interventions adhere to the MBT manual.

### Randomization

Copenhagen Trial Unit, a Danish center for clinical intervention research, will be responsible for the central randomization. Trial investigators will call designated staff at Copenhagen Trial Unit using a central telephone to randomize eligible participants to either the experimental group or the control group with a 1:1 allocation according to a computer-generated allocation sequence with permuted blocks of various sizes generated by Copenhagen Trial Unit and unknown to the investigators, secretaries, and clinical staff at the trial site. This is done to eliminate any predictability in the random sequence. The randomization is stratified by (1) sex and (2) high/low scores on the primary outcome measure, the Zanarini Rating Scale for Borderline Personality Disorder (ZAN-BPD) [38] at baseline.

### Interventions

The short-term MBT program delivered in this trial is overall similar to the existing long-term program, but differs structurally in the following ways: (1) the short-term program is lower in treatment intensity (both duration and exposure), (2) the same therapists provide both group and individual sessions in the short-term program (conjoined psychotherapy), whereas the group therapy and individual therapy are provided by different therapists in the long-term program (combined psychotherapy), and (3) the short-term program is structured in closed groups, in which all participants start and finish the program together, whereas the long-term program is structured as slow-open groups, in which a new participant can enter a group when another finishes. Both interventions in this trial adhere to the treatment guidelines provided by the National Institute for Health and Care Excellence [39].

#### Experimental intervention

The short-term MBT program is designed as a 20-week psychotherapy program consisting of five sessions of introductory MBT (MBT-I) followed by 15 sessions of group MBT (MBT-G) accompanied by conjoined individual sessions every second week and two psycho-educative meetings with participants and their relatives. Seven to nine participants and two therapists will be included in each short-term MBT group. The groups are closed to enhance cohesion between group participants. A total of 11 short-term MBT groups will be included in this trial.

Originally, MBT-I was a 12-session introductory psycho-educative program covering relevant topics like personality disorders, attachment, and mentalization [40]. The original manual has been modified for our 5-week intervention. A copy of our modified manual is available upon request. After the completion of MBT-I, the same group of participants will move on to MBT-G, which consists of 15 sessions of mentalization-based psychotherapy in groups, as manualized by Bateman and Fonagy [14]. In our short-term MBT program, group sessions will be accompanied by individual psychotherapy every second week with one of the two group therapists. As part of the individual therapy, a case formulation will be prepared and subsequently shared by the participants in the group. The overall purpose of the individual sessions is for the therapist and participant to develop a consensus of the participant’s main difficulties and to establish psychotherapeutic focus points in the group therapy. Furthermore, participants and relatives will be invited to two psycho-educative meetings hosted by the therapists at the beginning of the treatment program to enhance the mentalization work at home. The participants in the short-term MBT program will furthermore be offered three individual follow-up sessions after the end of treatment.

#### Control intervention

Long-term MBT is organized as a 14-month program and has been implemented at the clinic for the past 7 years. All participants randomized to long-term MBT will initially enter a 6-week MBT-I program manualized by Karterud and Bateman [40] and modified for our 6-week intervention in collaboration with the authors. New MBT-I groups commence every time new participants are recruited and randomized to long-term MBT. A maximum of 12 participants can enter an MBT-I group. When MBT-I finishes, participants will be allocated to one of eight slow-open MBT treatment groups. MBT-G is then organized as 12 months of weekly group therapy sessions, also manualized by Bateman and Fonagy [14]. In the long-term MBT program, group sessions will be accompanied by combined individual MBT sessions every 2 weeks throughout the program. As part of the individual therapy, a case formulation will be developed and subsequently shared with the group by the participant. Furthermore, participants and relatives will also be invited to two psycho-educative meetings hosted by the therapists to enhance the mentalization work at home. When a participant drops out or completes MBT-G, a new participant can start in the group. This procedure continues until the target sample size has been randomized to the long-term MBT program. The participants in the long-term MBT program will also be offered three individual follow-up sessions after the end of treatment.

#### Concomitant interventions

Participants who are receiving psychotropic treatment will be allowed to continue their medical treatment while participating in the trial. The medical protocol will follow national as well as international medical recommendations for the treatment of borderline personality disorder and comorbid disorders [39, 41]. Psychiatrists or attending physicians at the clinic will assess the need for additional psychotropic treatment and are asked to adhere to the guidelines. All participants, regardless of treatment condition, will be asked about their current medication by trial personnel during trial interviews to allow us to measure any potential differences in the use of psychotropic medication between the groups.

### Baseline assessment at trial intake

Baseline assessments will be carried out prior to randomization by the principal investigator, the sponsor-investigator, and a trained research assistant, all of whom are also clinical psychologists. *General psychopathology* will be assessed with the Mini International Neuropsychiatric Interview (MINI) [42]. *Personality disorders* will be assessed with the Structured Clinical Interview for DSM-V Personality Disorders (SCID-5-PD), formerly known as SCID-II [43]. The SCID-5-PD is considered the gold standard for clinician-administered semi-structured interviews designed to assess personality disorders according to DSM-V criteria [44].

### Outcomes

#### Primary outcome

The primary outcome is the severity of borderline symptomatology assessed with the ZAN-BPD [38], which is a clinician-administered scale for the assessment of change in borderline psychopathology over time. Each of the nine borderline personality disorder criteria are rated on a 0 to 4 anchored scale reflecting the severity of symptoms. The rating is intended to reflect both the frequency and the severity of borderline psychopathology. The interview provides a total score of borderline psychopathology ranging from 0 to 36. ZAN-BPD will be assessed by investigators blind to treatment allocation at baseline, and at the 8-, 16-, and 24-month follow-ups. We will video-record interviews to allow an assessment of inter-rater reliability based on the intraclass correlation coefficient. The results will be evaluated using the guidelines provided by Cicchetti [45].

#### Secondary outcomes

*Functional impairment* will be assessed with the Work and Social Adjustment Scale (WSAS) [46, 47]. This self-report scale will be assessed at baseline, and at the 8-, 16-, and 24-month follow-ups. *Quality of life* will be assessed with the Short-Form Health Survey (SF-36) [48], which consists of a mental and a physical component. We will use the mental component as a secondary outcome and the physical component as an exploratory outcome. This self-report scale will be given at baseline, and at the 8-, 16-, and 24-month follow-ups. *Global functioning* will be measured with the Global Assessment of Functioning (GAF) split version [49]. GAF will be assessed by investigators blind to treatment allocation at baseline, and at the 8-, 16-, and 24-month follow-ups. Inter-rater reliability will be calculated using the previously mentioned guidelines. *Severe self-harm* (dichotomous data) will be measured as the proportion of participants with severe self-harm defined as deliberate acts of self-harm resulting in visible tissue damage. Self-harm will be assessed by investigators blind to treatment allocation using the Suicide and Self-harm Inventory (SSHI) (citation) at baseline, and at the 8-, 16-, and 24-month follow-ups.

#### Exploratory outcomes

*Symptom distress* will be measured with the Global Severity Index (GSI) of the Symptom Checklist 90-R (SCL-90-R) [50]. SCL-90-R will be given at baseline, and at the 8-, 16-, and 24-month follow-ups.

### Potential predictors and mediators

Questionnaires are given at baseline and every fourth week throughout the intervention period for both intervention groups to allow us to explore predictors and mechanisms of change. In a separate statistical analysis plan, which will be submitted for publication before data collection is completed in this trial, we will describe how these exploratory analyses will be performed. The following predictors and mediators will be investigated.

*Personality functioning* will be assessed with the Levels of Personality Functioning Scale, Brief Form (LPFS-BF) [51], which is a newly developed brief 12-item self-report questionnaire assessing levels of personality functioning according to the DSM-V alternative model for personality disorders. *Attachment* will be assessed with the brief self-report Relationship Questionnaire (RQ), which gives continuous and categorical ratings of the four attachment styles [52]. *Borderline symptomatology* will be assessed using the Zanarini Rating Scale for Borderline Personality Disorder, Self-Report Version (ZAN-BPD-SRV) [53]. *Group alliance* will be assessed using the 12-item version of the Group Questionnaire (GQ) [54], which is a brief self-report measure of the three core components of group alliance: alliance to the other participants, alliance to the therapists, and group cohesion as a whole. *Mentalization skills* will be assessed with the 15-item Mentalization Questionnaire (MZQ) [55].

For an overview of all measures and the corresponding time of assessment, see Table 2.

**Table 2:** Assessments administered at baseline and each follow-up point throughout the trial

Assessment points	Self-report measures	Expert ratings
Baseline	LPFS-BF, RQ, SF-36, WSAS, SCL-90-R, MZQ	MINI, SCID-5-PD, ZAN-BPD, GAF, SSHI
Every 4 weeks	GQ, MZQ, ZAN-BPD-SRV	–
Follow-ups at 8, 16, and 24 months	SF-36, WSAS, SCL-90-R, MZQ	ZAN-BPD, SSHI, GAF

*GAF* Global Assessment of Functioning, *GQ* Group Questionnaire, *LPFS-BF* Level of Personality Functioning Scale, Brief Form, *MINI* Mini International Neuropsychiatric Interview, *MZQ* Mentalization Questionnaire, *RQ* Relationship Questionnaire, *SCID-5-PD* Structured Clinical Interview for DSM-V Personality Disorders, *SCL-90-R* Symptom Checklist 90-R, *SF-36* Short-Form Health Survey 36, *SSHI* Suicide and Self-Harm Inventory, *WSAS* Work and Social Adjustment Scale, *ZAN-BPD* Zanarini Rating Scale for Borderline Personality Disorder, *ZAN-BPD-SRV* Zanarini Rating Scale for Borderline Personality Disorder, Self-Report Version

### Blinding

Trial participants and therapists will not be blind to treatment allocation. This is due to the difficulties of implementing an efficient blinding procedure in psychotherapy trials. Baseline assessments will be done before allocation of participants, and the outcome assessments will be performed by blinded assessors. Participants will be instructed to withhold information of their allocation group when assessed. The statistical analyses will be conducted by blinded external statisticians from Copenhagen Trial Unit with the intervention groups coded as A and B. The steering committee will write and agree on two abstracts while the blinding is intact; one assuming the experimental intervention group is A and the control intervention group is B, and the other assuming the opposite. After this, the randomization code will be broken [56, 57].

### Participant discontinuation and withdrawal

Participants can withdraw from the trial at any time without giving a reason and without consequences for future treatment at the clinic. To secure data for the trial, a trial investigator will contact the participant and ask what aspects of the trial the participant wishes to withdraw from: (1) the trial intervention or control group, (2) the assessment interviews, or (3) use of already collected data in analyses. If the participant specifies that they wish to withdraw fully and thereby withdraw from all the points above, their data will be deleted and not used in any analysis. The trial investigator will encourage the participant to continue attending the follow-up assessments.

### Data management

The data in this trial will be collected using electronic case report forms developed in the data collection system REDCap. The system has been approved by the Danish Data Protection Agency and fulfills the requirements for data security. Data from the interviews will be entered directly into REDCap on a tablet, and all self-report measures will be collected from REDCap. For a detailed overview of outcome measures and data collection time points, see Table 2. The only source data are participants’ signed consent forms.

Student assistants employed in the research department but not otherwise involved in the trial will make sure that all self-report measures are sent to participants at the right times, and that the data are complete for all participants enrolled in the trial. The REDCap database has an integrated audit trail to document any access to and changes of the data. The validated data will be exported to SAS for further statistical analyses by statisticians from Copenhagen Trial Unit.

### Statistical plan and data analysis

#### Sample size

The sample size was determined by the predicted change in the primary outcome measure, ZAN-BPD. A 3.5-point superiority margin is considered to be the minimal important difference. Consistent with previous trials that have used ZAN-BPD as an outcome measure for a patient group like ours [24, 58], we expect a standard deviation of 8. With power set at 80% and alpha set at 5% two-tailed, a sample size of 83 participants is needed in each treatment group, corresponding to a total of 166 participants.

#### Statistical methods

All continuous outcomes will be assessed using linear regression and dichotomous outcomes will be analyzed using logistic regression. The analyses will be based on an intention-to-treat population and will primarily be adjusted for the baseline value of the outcome of interest and the stratification variables used in the randomization. We will secondly adjust all analyses for the following design variables: age (18–30 and 30–60) and functional impairment as assessed with the overall baseline GAF score (0–48 and 49–100) [34].

We will use a five-step procedure [59] to assess if the thresholds for statistical and clinical significance are crossed and we will handle missing data according to the procedure suggested by Jakobsen et al. [60]. A detailed statistical analysis plan will be published before the analyses commence, in which we will provide a detailed description of all primary, secondary, and exploratory analyses. All analyses will be performed blinded with the two intervention groups concealed as A and B.

#### Interim analyses

An external data safety monitoring committee will perform interim analyses when 50% of the data have been collected according to the good clinical practice guidelines of the International Council for Harmonisation of Technical Requirements for Pharmaceuticals for Human Use [61]. It will decide whether the trial should stop or carry on. Early stopping criteria will follow the recommendations of Jakobsen et al. [59].

## Discussion

This trial will provide evidence of the beneficial and harmful effects of short-term compared to long-term MBT for outpatients with subthreshold or diagnosed borderline personality disorder. To the best of our knowledge, short-term MBT has never been tested before. Gaining more information on how different lengths of treatment work for specific subtypes of patients may help to minimize the potential burden from long-term psychotherapy for some, while at the same time it may identify subtypes of patients for whom short-term psychotherapy is contraindicated. This knowledge may enhance the cost-effectiveness of treatment options for borderline personality disorder. Further, this trial may provide information on the potential predictors and mediators of treatment response.

The present trial has several strengths. First, it has a high degree of external validity because of the relatively inclusive eligibility criteria. Second, the methodology is based on CONSORT, and was predefined and described in detail before randomization began, including, e.g., blinding of all possible parties and implementation of a central randomization system both for generating an allocation sequence and for concealing allocation. Third, the implementation of systematic treatment fidelity ratings allows us to investigate treatment fidelity in both groups.

Our trial also has limitations. First, no systematic review of the effects of short-term compared to long-term psychotherapy for psychiatric disorders is currently available. As mentioned earlier, we are currently performing such a review. Second, the long-term MBT intervention, which is 14 months of treatment in this trial, diverges in intensity (both duration and exposure) from the original 18-month program [13]. This is due to the fixed length of the treatment packages, which have been implemented in the Danish mental health care system. Third, we cannot account for any potential confounding variables because of the structural differences between the groups: the short-term MBT program is closed and conjoined, whereas the long-term program is slow-open and combined.

### Dissemination policy

The Danish population will be informed of the trial as well as its final results through national media. The results of the trial will be presented at all outpatient clinics treating borderline personality disorder in the Mental Health Services, Capital Region of Denmark, by the principal investigator or sponsor-investigator. The final and interim results will be presented at national and international conferences. Further, associations for patients and relatives will be informed about the results of the trial and its future implications. The trial results will be written up by the steering committee and will be published in international peer-reviewed journals. The government of Denmark will be informed of the results before a press release is issued but will have no influence on the reporting of results.

### Trial status

The current protocol is version 1, dated 9 October 2018. The first participant was enrolled on 24 September 2018. Recruitment is expected to be completed by September 1, 2020

## Additional file

Additional file 1: SPIRIT checklist. (DOCX 61 kb)

## Abbreviations

CONSORT: Consolidated Standards of Reporting Trials
DSM-V: Diagnostic and Statistical Manual of Mental Disorders, 5th edition
GAF: Global Assessment of Functioning
GQ: Group Questionnaire
GSI: Global Severity Index
LPFS-BF: Levels of Personality Functioning Scale, Brief Form
MBT: Mentalization-based therapy
MBT-G: Mentalization-based group therapy
MBT-I: Introduction to Mentalization-Based Therapy
MINI: Mini International Neuropsychiatric Interview
MZQ: Mentalization Questionnaire
RQ: Relationship Questionnaire
SCID-5-PD: Structured Clinical Interview for DSM-V Personality Disorders
SCL-90-R: Symptom Checklist 90-R
SF-36: Short-Form Health Survey 36
SPIRIT: Standard Protocol Items: Recommendations for Interventional Trials
SSHI: Suicide and Self-Harm Inventory
WSAS: Work and Social Adjustment Scale
ZAN-BPD: Zanarini Rating Scale for Borderline Personality Disorder
ZAN-BPD-SRV: Zanarini Rating Scale for Borderline Personality Disorder, Self-Report Version
